# Student Perspective: Internship Exploring Intellectual and Social Benefits of Adult Study Abroad

**DOI:** 10.1093/geroni/igab046.2219

**Published:** 2021-12-17

**Authors:** Kim Redlich

**Affiliations:** Georgetown University, Washington DC, District of Columbia, United States

## Abstract

Older adult participation in lifelong learning programs – such as university continuing education opportunities and the Age-Friendly University global network – has grown steadily over the last few years. Many of these programs are characterized by mixed-age classrooms in which undergraduate students share space and learning, remotely during Covid, with older adult participants who pay a nominal fee. Survey findings will be presented from older students involved in two university programs in this category: Temple University’s “senior scholars” program and Georgetown University’s “senior auditors” program, specifically related to the concept of adult study abroad. Adult study abroad is a new offering that combines the intellectual and social benefits of stimulating coursework with the transformative power of travel, and how the merging of these pursuits can produce purpose, meaning and community, especially for older adults. It is typically residential, academic, intergenerational, and of longer duration than a typical tourist experience.

